# Bendability optimization of flexible optical nanoelectronics via neutral axis engineering

**DOI:** 10.1186/1556-276X-7-256

**Published:** 2012-05-15

**Authors:** Sangmin Lee, Jang-Yeon Kwon, Daesung Yoon, Handong Cho, Jinho You, Yong Tae Kang, Dukhyun Choi, Woonbong Hwang

**Affiliations:** 1 Department of Mechanical Engineering, Pohang University of Science and Technology, San 31, Hyoja, Namgu, Pohang, Gyungbuk, 790-784, Republic of Korea; 2 School of Integrated Technology, Yonsei University, 162-1, Songdo-dong Yeonsu-gu, Incheon, 406-840, Republic of Korea; 3 Department of Mechanical Engineering, College of Engineering, Kyung Hee University, 1 Seocheon-dong, Giheung-gu, Yongin-si, 446-701, Republic of Korea

**Keywords:** Flexible optical nanoelectronics, Bendability optimization, Neutral axis engineering, Buffer layer

## Abstract

The enhancement of bendability of flexible nanoelectronics is critically important to realize future portable and wearable nanoelectronics for personal and military purposes. Because there is an enormous variety of materials and structures that are used for flexible nanoelectronic devices, a governing design rule for optimizing the bendability of these nanodevices is required. In this article, we suggest a design rule to optimize the bendability of flexible nanoelectronics through neutral axis (NA) engineering. In flexible optical nanoelectronics, transparent electrodes such as indium tin oxide (ITO) are usually the most fragile under an external load because of their brittleness. Therefore, we representatively focus on the bendability of ITO which has been widely used as transparent electrodes, and the NA is controlled by employing a buffer layer on the ITO layer. First, we independently investigate the effect of the thickness and elastic modulus of a buffer layer on the bendability of an ITO film. Then, we develop a design rule for the bendability optimization of flexible optical nanoelectronics. Because NA is determined by considering both the thickness and elastic modulus of a buffer layer, the design rule is conceived to be applicable regardless of the material and thickness that are used for the buffer layer. Finally, our design rule is applied to optimize the bendability of an organic solar cell, which allows the bending radius to reach about 1 mm. Our design rule is thus expected to provide a great strategy to enhance the bending performance of a variety of flexible nanoelectronics.

## Background

There has been rapid development in the field of flexible optical nanoelectronics such as organic solar cells (OSCs) and organic light-emitting diodes for future portable and wearable electronic nanodevices, which have potential personal and military applications [1-10]. These optical nanodevices basically require an optically transparent window to absorb or emit light. Indium tin oxide (ITO) thin films have been widely used as transparent electrodes for such optical nanoelectronics because of their high visible transparency, chemical stability, and excellent adhesion to a substrate [11-13]. However, despite its advantages, ITO is still difficult to apply to flexible optical nanodevices without damaging the electronic functionality under an external bending load because of its brittleness. Researchers are thus trying to find substitutes for ITO such as carbon nanotube, graphene, and aluminum-doped zinc oxide (AZO) [10,12-14]. However, with these alternatives, it is still difficult not only to successfully achieve a high-quality and low-cost production that is as good as ITO with high transparency (higher than 90 %) and low electric resistance (less than 10 Ω), but also to successfully increase bendability due to their brittleness which is common with ITO. Thus, it is critically necessary to develop innovative ideas and solutions to enhance mechanical stability of ITO under external bending loads.

To improve the bendability of flexible nanoelectronics, a buffer layer has been adopted [3,6,9,15]. Researchers have reported that the mechanical bendability of electronic nanodevices can be increased by using a buffer layer above or below the ITO layer. However, they did not suggest an optimized design rule that considers both the thickness and elastic modulus of the buffer material. Because various buffer layers could be used to increase the thermal, chemical, and mechanical stabilities of flexible electronic nanodevices, a governing design rule is crucially needed to optimize the bendability of these flexible nanodevices regardless of the buffer layers that are chosen. In this article, we report a design rule for the bendability optimization of flexible optical nanoelectronics through controlling the neutral axis (NA). If we place the fragile layer such as ITO in a nanodevice at the NA position, the bending stress and strain in the layer are greatly reduced, thus enhancing the bendability of the device. Therefore, we first investigate the behavior of the NA position and the effect on the device bendability by independently considering the elastic modulus and thickness of a buffer layer on the ITO. Because the elastic modulus and thickness of a buffer layer influenced each other when determining the NA, we should consider these parameters together. Therefore, we develop a design rule for the bendability optimization of flexible electronics by controlling the NA position, considering both the thickness and elastic modulus of the buffer layer. Finally, our design rule to optimize the bendability of flexible devices is applied to an inverted OSC with an ITO optical window. We believe that our design rule based on NA engineering will provide a great advantage to improve the bendability of flexible nanoelectronics.

## Methods

### Bending theory

**Figure 1:**
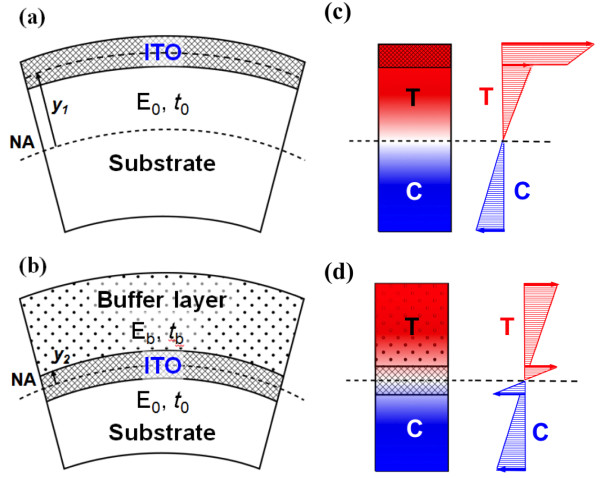
** Scheme for NA positions of a flexible film including a brittle material (here, ITO).** (**a**) Without and (**b**) with a buffer layer. By adopting a buffer layer, NA can be controlled to be located to the brittle material, leading to high bendability. (**c**, **d**) Illustrations of corresponding stress distributions for (a) and (b), respectively. (E: elastic modulus, *t*: thickness, T: tensile stress, C: compressive stress).

Figure 1 shows a cross-sectional illustration of the stress distribution of an ITO film by controlling NA. The position of the NA is determined from the condition that the resultant axial force acting on the cross section is zero:

(1) $$\int_{A}\sigma_{A}dA=0$$

where *σ* is the longitudinal stress and *dA* is the element of cross-sectional area. The strain (*ϵ*) curvature relation in pure bending is

(2) $$\epsilon =-\frac{y}{\rho }=-\kappa y$$

where *y* is the distance from the NA, *ρ* is the bending radius, and *κ* is the curvature. The most common stress–strain relationship encountered in engineering is the equation for a linearly elastic material. For such materials, we substitute Hooke's law for uniaxial stress into Equation 2 and obtain

(3) σ=Eϵ=−Eκy

In this equation, the normal stresses acting on the cross section are proportional to the distance, *y*, from the NA at a given *κ*. Thus, it is important to locate the most fragile material at the NA position to minimize the stress applied to the material. As shown in Figure 1a,b, when there are homogeneous films with a symmetric cross section, such as ITO and a substrate, we compared the maximum bending stresses of ITO films without and with a buffer layer. Based on Equation 3, the maximum bending stress of the ITO with a buffer layer can be significantly decreased more than that of the ITO without a buffer layer because $y_{2}<<y_{1}$, as shown in Figure 1c,d. In other words, by adopting a buffer layer, a fragile layer such as ITO can be located at the NA position and the bending stress acting on the fragile layer can be greatly reduced, thus leading to flexible nanoelectronics with high bendability.

## Results and discussion

**Figure 2:**
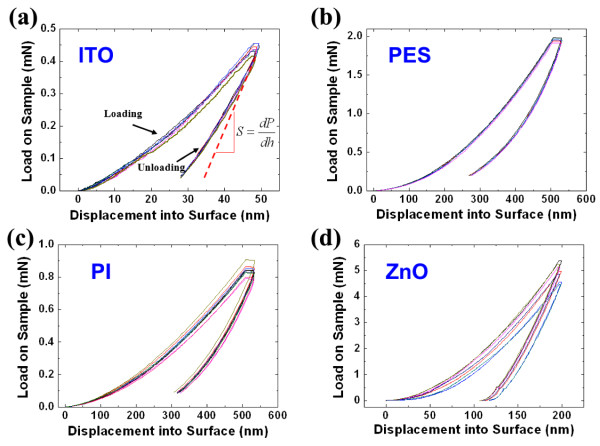
** Load-displacement data for depth-sensing indentation experiment.** (**a**) ITO, (**b**) PES, (**c**) PI, (**d**) ZnO. (*S*: contact stiffness, *P*: load, *h*: distance).

To investigate the bendability of a nanodevice according to the NA position, the elastic properties for each material should be determined. The elastic modulus of thin layers can be measured by using standard nano-indentation techniques. These techniques depend on the fact that the displacements recovered during unloading are largely elastic, which allows the Oliver and Pharr method to be used to determine the modulus from an analysis of indentation load–displacement data [16-19]. The load (*P*) and displacement (*h*) are measured directly from the indentation load–displacement curve, as shown in Figure 2a, and the contact stiffness (*S*) is determined by measuring the unloading slope at peak load. If the values are determined, we can obtain the elastic modulus for each material by using the analysis procedure of the Oliver and Pharr method [16]. Four specimens, including ITO, polyethersulfone (PES), polyimide (PI), and zinc oxide (ZnO), were prepared as listed in Table 1, and nano-indentation tests for each material were performed under displacement control with maximum displacements from 50 to 500 nm using an MTS nanoindenter XP system with a Berkovich diamond indenter (Eden Prairie, MN, USA). The tests were conducted at least 10 times for each specimen. Figure 2 shows the load-indentation data for a depth-sensing indentation experiment. The measured elastic moduli for each material are shown in Table 1, and these values are similar to the previous results [6,20,21]. The Poisson ratios of each material were obtained from the results of previous researches [22-25].

**Table 1:** Mechanical properties of ITO, PES, PI, and ZnO

**Material**	**ITO**	**PES**	**PI**	**ZnO**
Thickness (nm)	80	200 × 10^3^	50 × 10^3^	520
Modulus (GPa)	116	4.6	2.0	96.1
Standard deviation	3.69	0.03	0.07	3.3
Poisson ratio	0.25	0.42	0.35	0.25

**Figure 3:**
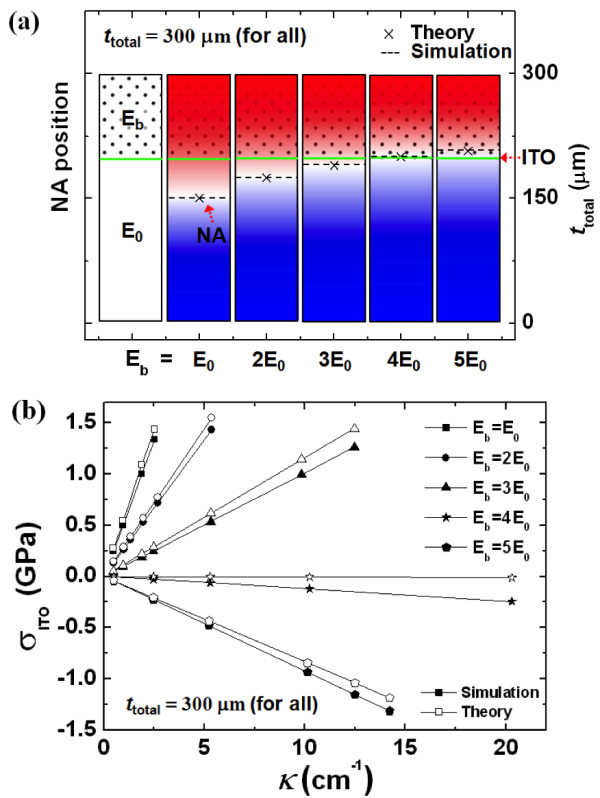
** Behavior of NA position only by considering*E***_**b**_**.** (**a**) NA position depending on *E*_b_ by the NA theory and simulation (red: tension, blue: compression). (**b**) Maximum stress of ITO layer according to *κ* for various *E*_b_.

We investigated how the NA position varied with the elastic modulus and thickness of the buffer layer. First, we considered the elastic modulus of the buffer layer (*E*_b_). We assumed an 80-nm-thick ITO layer on a PES substrate with an elastic modulus of *E*_0_ and a thickness of 200 μm. When a 100-μm-thick buffer layer was placed on the ITO layer, we calculated the NA position using the NA theory from Equations 1 and 3, and along with a simulation using an ABAQUS 6.10 simulator (Dassault Systèmes Simulia, Providence, RI, USA) with various values for *E*_b_. The calculated results from the two methods were about the same, as shown in Figure 3. The NA position gradually became closer to the ITO layer as *E*_b_ increased, and they closely corresponded when *E*_b_ became four times *E*_0_, as shown in Figure 3a. Then, when *E*_b_ became larger than 4 *E*_0_, the NA position separated from the ITO layer again. In other words, when the thickness of the buffer layer was selected, a specific *E*_b_ made it possible to position the ITO layer at the NA. Figure 3b shows the corresponding maximum stress of the ITO layer according to the bending curvature (*κ*) for various *E*_b_. When *E*_b_ is equal to *E*_0_, tensile stress in the ITO layer occurred because the NA position was located below the ITO layer, and this tensile stress gradually decreased as *E*_b_ increased to 4 *E*_0_. Then, when *E*_b_ was larger than 4 *E*_0_, compressive stress in the ITO layer occurred because the NA position was located above the ITO layer, and this compressive stress increased proportionally as *E*_b_ increased over 4 *E*_0_. In other words, the maximum stress on the ITO decreased proportionally as *E*_b_ increased up to 4 *E*_0_ because the NA position was gradually closer to the ITO layer, and this effect brought about the results that increased the critical bending curvature (i.e., the smallest radius that the layers can be bent before the critical bending radius decreased, which is the fracture). In particular, in the case of *E*_b_ = 4 *E*_0_, when the maximum bending curvature was 20 cm^−1^ (i.e., maximum bending radius was 500 μm), the maximum stress was only about 250 MPa. Considering the yield strength of 1.2 GPa for ITO [26], it would not lose its inherent properties up to a critical bending radius of 100 μm. In Figure 3, the slight difference in the results between the theory and simulation was probably caused by the assumption that a cross-sectional area of the beam in the theory does not change when being bent (i.e., the theory does not consider the effect of Poisson's ratio).

**Figure 4:**
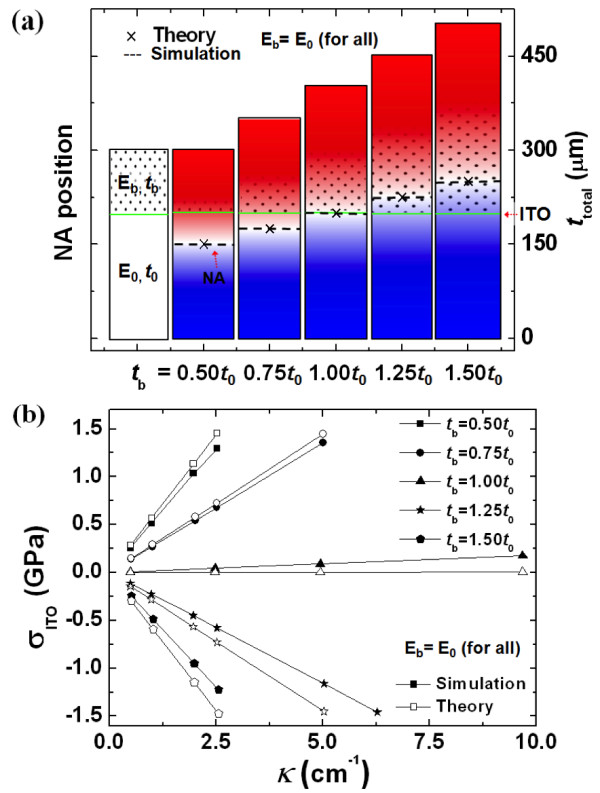
** Behavior of NA position only by considering the*t***_**b**_**of a buffer layer.** (**a**) NA position depending on *t*_b_ by the NA theory and the simulation (red: tension, blue: compression). (**b**) Maximum stress of ITO layer according to *κ* for various *t*_b_.

**Figure 5:**
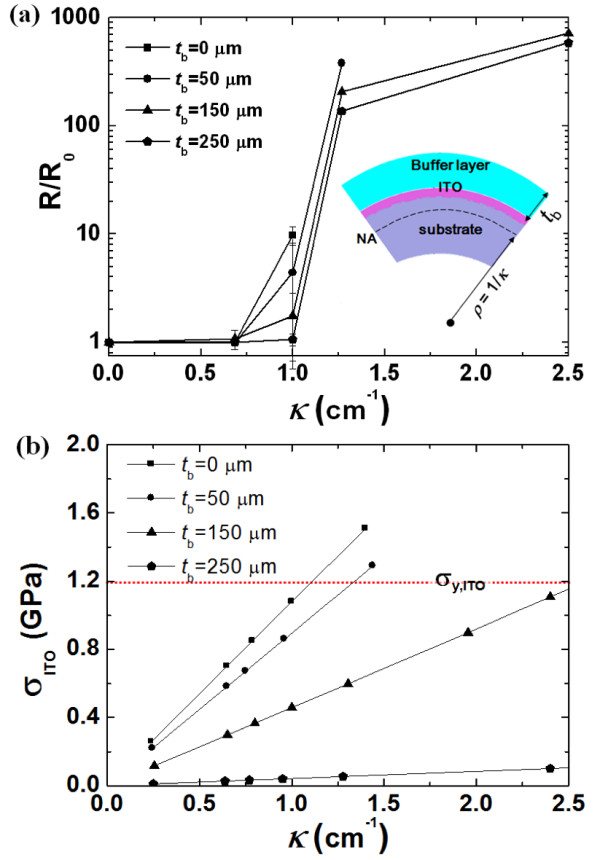
** Experimental verification of enhanced bendability of ITO-based thin films according to the*t***_**b**_**of PI.** (**a**) Two-probe resistance and (**b**) maximum stress of ITO layer for PI/ITO/PES on bending curvature *κ* (*σ*_y_: yield strength).

Next, we considered the effect of the thickness of the buffer layer (*t*_b_) on the NA, as shown in Figure 4. We assumed that an 80-nm-thick ITO layer was on a PES substrate with *E*_0_ and a thickness of 200 μm. When *E*_b_ was the same as *E*_0_, the NA position was calculated with various values for *t*_b_ using the two methods mentioned above. When *t*_b_ was lower than *t*_0_, the NA position was located below the ITO layer, and the tensile stress in the ITO layer occurred. The NA position became closer to the ITO layer as *t*_b_ increased up to *t*_0_, and this corresponding tensile stress gradually decreased. Then, when *t*_b_ was larger than *t*_0_, the ITO layer was located above the NA position, and the compressive stress in the ITO layer occurred. This compressive stress increased proportionally as *t*_b_ increased over *t*_0_, as shown in Figure 4a. In the case of *t*_b_ = *t*_0_, considering the yield strength of ITO, the critical bending curvature was about 60 cm^−1^ (i.e., the bending radius was about 167 μm), as shown in Figure 4b. Thus, by controlling the elastic modulus and thickness of a buffer layer, the NA position could be easily controlled, and the mechanical bending stability of the ITO layer could be significantly increased when the ITO layer was located at the NA position. In order to confirm the theoretical behavior of the NA and the corresponding mechanical durability, we performed an experiment in which the electrical resistance of ITO films was measured as the bending curvature increased. PI was adopted as a buffer layer on an ITO film (see the inset in Figure 5a), and we increased the *t*_b_ of PI from 0 to 250 μm. The PI thickness was simply controlled using a PI adhesive tape with a thickness of 50 μm, and we confirmed that the elastic modulus of the PI film does not change by the nano-indentation test when the thickness increased from 50 to 250 μm. Figure 5a shows the dependence of the two-probe resistance for PI(*t*_b_)/ITO/PES on bending curvature *κ*, and *R*/*R*_0_ indicates the bent-to-unbent ratio of the two-probe resistance. The initial curvature of all the structures was almost flat up to 0.7 cm^−1^. When the curvature was higher than 0.7 cm^−1^, the resistance for all of the structures increased. As the thickness of the buffer layer increased, the change in resistance decreased. In particular, in the case of a buffer layer with a thickness of 250 μm, the resistance was almost zero up to a curvature of 1 cm^−1^. Then, the resistances for structures with buffer layer thicknesses of 0, 50, and 150 μm could not be obtained for curvatures of 1, 1.2, and 2.5 cm^−1^, respectively. This was caused by a fracture of the ITO layer in the structures. Thus, we confirmed that the bendability of the ITO film could be increased by employing a buffer layer, and this was because the maximum bending stress of the ITO layer was decreased by locating the ITO layer near the NA position based on Equation 3.

Again, to confirm these results, we performed a simulation of the PI(*t*_b_)/ITO/PES structure to determine how the maximum bending stress of ITO changed with the buffer layer thickness. Based on the simulated results, we found when the fracture of the ITO layer occurred for each specimen and compared the simulated results with the experimental results. As shown in Figure 5b, the yield of ITO layers consisting of a buffer layer with thicknesses of 0, 50, and 150 μm respectively occurred at curvatures of about 1.1, 1.3, and 2.6 cm^−1^, and these results were in close agreement with those of the experiment. However, the structures with a buffer layer showed relatively high-level resistances when the bending curvature was larger than 1 cm^−1^ (i.e., *R*/*R*_0_ were greater than 100). This might be caused by the imperfect bonding of the buffer layer because the PI adhesive tapes were not firmly bound to one another when the thickness was increased. Even though the structures with a buffer layer showed relatively high resistance, for the above-mentioned reason, the simulation and experiment demonstrated that ITO-based structures with a buffer layer are highly reliable and durable during bending. As stated earlier, to significantly increase the bendability of flexible devices, we should place the ITO layer at the NA position.

**Figure 6:**
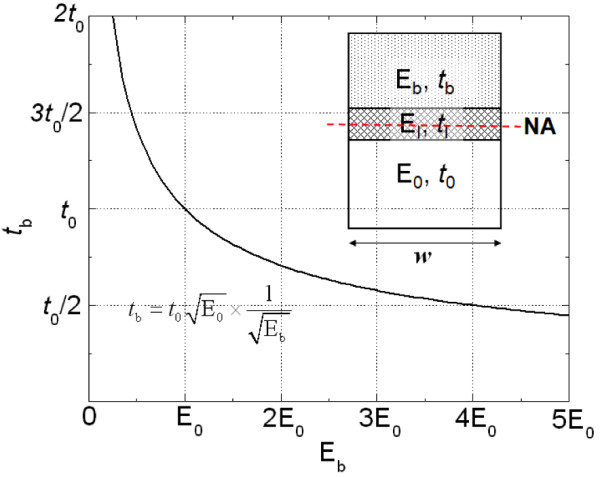
** Relationship between*E***_**b**_**and*t***_**b**_**.** Considering a simple multilayer model composed of three kinds of materials including substrate (*E*_0_, *t*_0_), ITO (*E*_I_, *t*_I_), and buffer layer (*E*_b_, *t*_b_).

So far, although we independently considered the effects of *E*_b_ and *t*_b_, when the other was fixed, all of them dependently had an influence on the NA position. Thus, it is necessary to develop the governing design rule for the bendability optimization, considering the relationship between them. To deduce this relationship, we considered a simple multilayer model composed of three kinds of materials, including the substrate (*E*_0_, *t*_0_), ITO (*E*_I_, *t*_I_), and buffer layer (*E*_b_, *t*_b_), as shown in Figure 6 (inset). From Equations 1 and 3, the equation for locating the NA becomes

(4) $$E_{\text{b}}\int_{\text{b}}ydA+E_{I}\int_{I}ydA+E_{0}\int_{0}ydA=0$$

Assuming that the NA is at the center of the ITO, this equation can be obtained by substituting the expressions for *y* and *A* into the expressions for *t* and *w*; thus,

(5) $$E_{\text{b}}\left(\frac{t_{\text{b}}+t_{I}}{2}\right)t_{\text{b}}w+E_{I}\left(0\right)t_{\text{b}}w-E_{0}\left(\frac{t_{0}+t_{I}}{2}\right)t_{0}w=0$$

where *A* and *w* are the cross-sectional area and width, respectively. Because the ITO layer for optical electronics is very thin ($t_{\text{I}}<<t_{0}$, *t*_b_), we can set *t*_I_ to zero. Thus, the relationship between *E*_b_ and *t*_b_ is as follows:

(6) $$E_{\text{b}}t_{\text{b}}^{2}=E_{0}t_{0}^{2}$$

Equation 6 can be solved for *t*_b_ in terms of *E*_b_, obtaining the following relationship:

(7) $$t_{\text{b}}^{}=t_{0}^{}\sqrt{E_{0}}\times \frac{1}{\sqrt{E_{\text{b}}}}$$

This equation shows that *t*_b_ is inversely proportional to the square root of *E*_b_, as shown in Figure 6. This equation provides the design rule that can optimize the bendability of flexible optical nanoelectronics by tuning both the elastic modulus and thickness of a buffer layer. In other words, when designing flexible optical electronics, we can choose a specific material of any thickness and improve not only the mechanical bendability, but also the electrical efficiency. Because there is an enormous variety of materials and structures in flexible electronics, our design rule can have a great effect on these devices. Even though we assumed that one fragile layer exists between the substrate and buffer layer, this design rule can be applied to optical nanoelectronics with multiple fragile layers such as conductive oxides, including ITO, ZnO, and AZO, because the thickness of the conductive oxides for optical electronics is typically less than a few hundred nanometers and all fragile layers are located near the NA. Although the multiple layers exist in OSCs, their effect in Equation 5 is very small and is negligible. Thus, we can obtain the optimized thickness of each buffer layer from Equation 7 when the buffer layers with various material properties are respectively employed.

**Figure 7:**
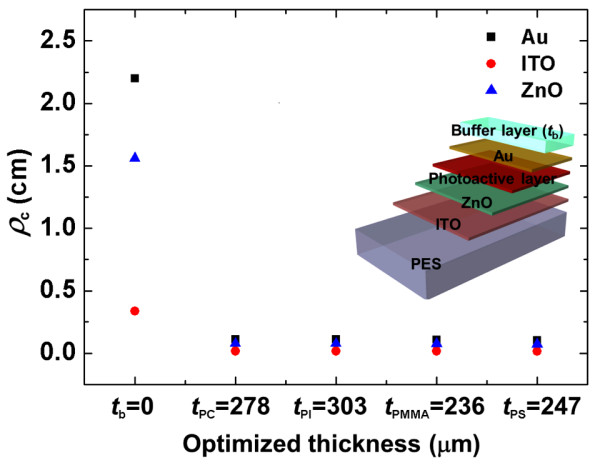
** Critical bending radius for inverted OSCs.** Composed of various buffer materials with thickness optimized by the design rule based on NA engineering.

To demonstrate our design rule for the bendability optimization of flexible electronics with multiple fragile layers, we applied different buffer materials (polycarbonate (PC), PI, poly(methyl methacrylate) (PMMA), and polystyrene (PS)) to inverted OSCs with an ITO optical window. The inverted OSC was composed of five layers, including PES (200 μm), ITO (80 nm), ZnO (50 nm), poly(3-hexylthiophene)/[6,6]-phenyl-C_61_-butyric acid methyl ester blend photoactive layer (*E* = 6.02 GPa, *ν* = 0.35, *t* = 100 nm), and Au (*E* = 69.8 GPa, *ν* = 0.44, *t* = 70 nm) [27,28]. To optimize the bendability of flexible electronics, the thickness of each buffer material was calculated based on the elastic modulus of buffer layers from Equation 7. The optimized thicknesses of PC, PI, PMMA, and PS were 278, 303, 236, and 247 μm, respectively. Figure 7 shows the critical bending radius (*ρ*_c_) for inverted OSCs with different buffer materials from the simulation, considering the yield strength for each material (*σ*_y_ = 200 MPa for ZnO and *σ*_y_ = 120 MPa for Au) [29]. While the *ρ*_c_ of the device with no buffer layer was only about 2.2 cm due to a fracture of Au, the *ρ*_c_ of each device with the buffer material, including optimized PC, PI, PMMA, and PS, were 1.11, 1.11, 1.09, and 1.05 mm, respectively. All of the inverted OSCs with different buffer materials to which our design rule was applied had almost the same *ρ*_c_, which was at least 20 times lower than that with no buffer layer. These results clearly showed that our design rule could optimize the bendability of flexible nanoelectronics with various buffer materials. Further, the *ρ*_c_ of each component in the device without a buffer layer such as Au, ITO, and ZnO varied from 2.2 to 0.34 cm, depending on their material properties, whereas in the case of the device with a buffer layer, *ρ*_c_ values of the components were on a similar level (i.e., low *ρ*_c_), regardless of their properties. This was because all of the thin fragile components were located near the NA position, and this result clearly showed that our design rule can also be applied to flexible optical nanodevices with multiple thin, fragile layers. In other words, no matter what buffer materials and thin components are used in the devices, the bendability can be significantly enhanced by the design rule based on NA engineering. Thus, this design rule could provide valuable guidance for the design and performance optimization of flexible optical nanoelectronics.

## Conclusions

In summary, we have clearly demonstrated that the bendability of flexible optical nanodevices could be significantly enhanced by NA engineering, considering both the thickness and elastic modulus of a buffer layer on the ITO layer. Because the material property and geometry of a buffer layer could be different based on the purpose of a flexible electronic nanodevice, our design rule, which considers both the thickness and modulus of a buffer layer, is anticipated to be suitable for the bendability optimization of various flexible nanoelectronics. Furthermore, our design rule was applied to inverted OSCs with various buffer materials, and we confirmed that all of the OSCs showed excellent bendability, whatever buffer materials were chosen. Thus, our strategy may provide a wide range of opportunities for a variety of flexible electronic applications.

## Competing interests

The authors declare that they have no competing interests.

## Authors’ contributions

SL and J-YK equally contributed to this work. WH and DC conceived the initial idea and designed the schemes. SL, J-YK, and DY performed the numerical simulations, and HC and JY performed the experiments. WH, DC, J-YK, and YTK, with partial contributions from SL, characterized the results. DC and SL wrote the paper with partial contributions of WH and J-YK. All authors discussed the results, commented on the manuscript, and approved the final manuscript.
